# How to estimate complementarity and selection effects from an incomplete sample of species

**DOI:** 10.1111/2041-210X.13285

**Published:** 2019-08-14

**Authors:** Adam Thomas Clark, Kathryn E. Barry, Christiane Roscher, Tina Buchmann, Michel Loreau, W. Stanley Harpole

**Affiliations:** 1 Department of Physiological Diversity, Helmholtz Centre for Environmental Research (UFZ), Leipzig, Germany; 2 German Centre for Integrative Biodiversity Research (iDiv) Halle-Jena-Leipzig, Leipzig, Germany; 3 Synthesis Centre for Biodiversity Sciences (sDiv), Leipzig, Germany; 4 Institute of Biology, Leipzig University, Leipzig, Germany; 5 Department of Community Ecology, Helmholtz Centre for Environmental Research (UFZ), Halle, Germany; 6 Centre for Biodiversity Theory and Modelling, Theoretical and Experimental Ecology Station, CNRS, Moulis, France; 7 Institute of Biology, Martin Luther University Halle-Wittenberg, Halle (Saale), Germany

**Keywords:** biodiversity, community ecology, conservation, ecosystem functions, monitoring, partitionBEFsp, sampling, statistics

## Abstract

1. Declines in global biodiversity have inspired a generation of studies that seek to characterize relationships between biodiversity and ecosystem functioning. The metrics for complementarity and selection effects derived by Loreau and Hector in 2001 remain some of the most influential and widely used statistics for studying these relationships. These metrics quantify the degree to which the effect of biodiversity on a given ecosystem function depends on only a few species that perform well in monoculture and in mixture (the selection effect) or if the effect of biodiversity on a given ecosystem function is independent of monoculture performance (the complementarity effect). This distinction may be useful in determining the consequences of the loss of rare versus common or dominant species in natural systems. However, because these metrics require observations of all species in a community in monoculture, applications in natural systems have been limited.
2. Here, we derive a statistical augmentation of the original partition, which can be applied to incomplete random samples of species drawn from a larger pool. This augmentation controls for the bias introduced by using only a subsample of species in monocultures rather than having monocultures of all species.
3. Using simulated and empirical examples, we demonstrate the robustness of these metrics, and provide source code for calculating them. We find that these augmentations provide a reliable estimate of complementarity and selection effects as long as approximately 50% of the species present in mixture are present in monoculture and these species represent a random subset of the mixture.
4. We foresee two primary applications for this method: (a) estimating complementarity and selection effects for experimentally assembled communities where monoculture data are lacking for some species, and (b) extrapolating results from biodiversity experiments to diverse natural systems.

## 1 Introduction

Global biodiversity is declining at unprecedented rates (Newbold et al., 2015; Tittensor et al., 2014). These large-scale declines have inspired a generation of experiments that measure effects of species loss on local ecosystem properties such as biomass production, carbon sequestration, nutrient cycling and trophic interactions (Barry et al., 2019; Cardinale et al., 2012, 2006; Cardinale, Hillebrand, Harpole, Gross, & Ptacnik, 2009; Tilman, Isbell, & Cowles, 2014). The primary result from this body of research has been that the loss of randomly selected species at local scales generally results in declines in ecosystem functioning, especially for commonly measured functions such as above-ground biomass production (but see Chen et al., 2017; Meyer et al., 2016).

Problematically, experiments may be limited in their capacity to predict the consequences of species loss in naturally assembled systems (van der Plas, 2019). First, declines in biodiversity are often not detected at local scales in natural systems (Vellend et al., 2013; Dornelas et al., 2014; but see Gonzalez et al., 2016), potentially indicating that species losses are compensated for by invasions and range expansions. In contrast, experiments are typically managed to prevent immigration (Fargione, Brown, & Tilman, 2003; Leibold & Chase, 2018). Second, when species are lost from naturally assembled systems, rare species are far more likely to go extinct than are common and dominant species (Davies, Margules, & Lawrence, 2004; Kunin & Gaston, 1997), which may impact systems differently than the randomized species loss that is implemented in most experiments (Barry et al., 2019; Schlapfer, Pfisterer, & Schmid, 2005; Smith & Knapp, 2003). Third, in experiments, abiotic variation is usually minimized in order to accurately measure direct effects of diversity. However, these controls also dampen effects of abiotic factors that may be important in natural systems, such as nutrient or water availability (Diaz et al., 2007; Grace et al., 2016). In spite of the limited predictive capacity of biodiversity–ecosystem functioning experiments, many common metrics for measuring biodiversity–ecosystem functioning relationships cannot be applied to naturally assembled systems.

The most common metrics for biodiversity–ecosystem functioning relationships quantify the effect of biodiversity on plant productivity in terms of ‘relative yield’ – i.e. the ratio of species-level productivity observed in a multi-species mixture, relative to that in monoculture (Trenbath, 1974; de Wit, 1960). Observed relative yield is then compared to ‘expected relative yield’, which is usually assumed to be proportional to initial relative abundance (e.g. the proportion planted or sown). If observed relative yield exceeds expected relative yield, then productivity per unit area is higher in mixtures than in the average monoculture. This comparison provides an intuitive ‘baseline’ for subsequent tests and relates directly to the practical question: ‘if my goal is to maximize productivity, am I better off planting many small monocultures, or a single large mixture?’. A major limitation that hinders applications of these metrics in naturally assembled systems is that they require information about species-level functioning observed both in monoculture and in mixture. While monocultures of all species in the community may be available in some biodiversity experiments (Potvin & Gotelli, 2008; Roscher et al., 2004; Tilman et al., 1997), similar data are generally not available for natural communities (Mori, 2018).

An advantage of metrics based on relative yield is that they can be decomposed into partitions that additively summarize different aspects of biodiversity–ecosystem functioning relationships (Fox, 2006; Isbell et al., 2018; Loreau & Hector, 2001). By far the most widely used of these partitions, hereafter the Loreau and Hector partition, groups deviations between observed and expected yield into two components: the ‘selection effect’ and the ‘complementarity effect’ (Loreau & Hector, 2001). The selection effect, calculated as the covariance between monoculture yield (*M*) and deviation in relative yield (Δ*RY*), quantifies deviations that are associated with species performance in monoculture. The complementarity effect is the remaining difference in yield and summarizes deviations that are statistically independent of monoculture performance. While the Loreau and Hector partition does not necessarily identify specific mechanisms behind these deviations, it can provide useful insight into how species loss may affect ecosystem functioning (e.g. loss of rare vs. dominant species) (Barry et al., 2019; Loreau & Hector, 2001; Loreau, Sapijanskas, & Hector, 2012; Turnbull, Isbell, Purves, Loreau, & Hector, 2016). Although the Loreau and Hector partition is primarily used in grassland ecosystems that are dominated by perennials (reviewed by Barry et al., 2019; Hector et al., 2009; Tilman et al., 2014), it has also been successfully applied in experiments in subtropical and temperate forests (e.g. Ferlian et al., 2018; Huang et al., 2018), short-lived annual plant communities (e.g. Roscher & Schumacher, 2016), and marine macroalgal communities (e.g. Bruno, Boyer, Duffy, Lee, & Kertesz, 2005; Bruno et al., 2006).

**Figure 1:**
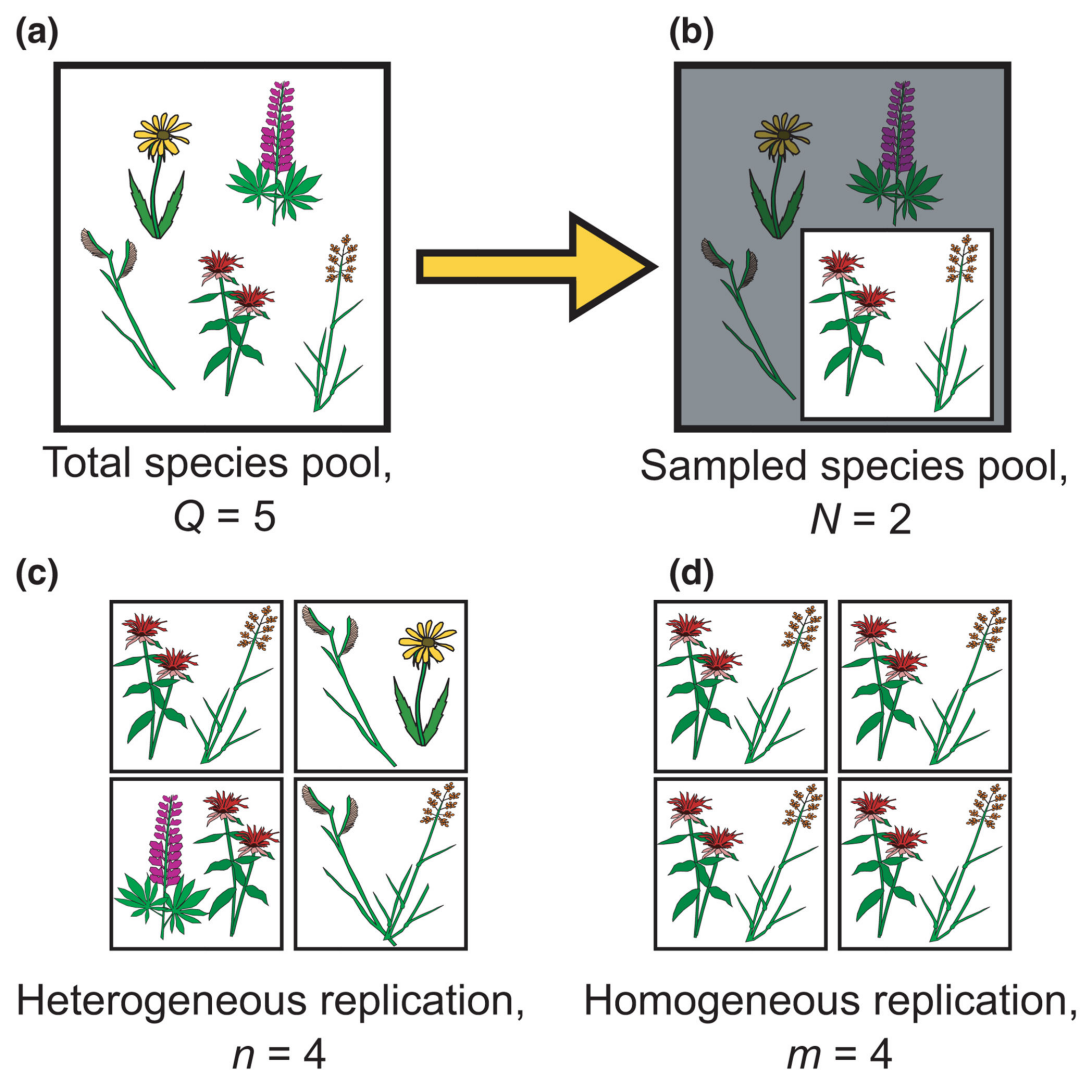
Examples of sampling (a–b) and replication (c–d) schemes, following the notation used in the main text. (a) The total species pool describes the full set of species that are present in a community. Here, the community includes five species (i.e. *Q* = 5). (b) The sampled community describes species from the total community that have actually been sampled or observed. Here, two of five total species have been sampled, shown in the white square (i.e. *N* = 2). (c) Heterogeneous replication refers to repeated measurements of distinct groups of randomly chosen species. Here, we have drawn four randomly chosen 2-species communities (i.e. *n* = 4). (d) Homogeneous replication refers to repeated measurements of the same community, as is typically applied in experiments. Here, we sample the same community of two species four times (i.e. *m* = 4)

Because the classic Loreau and Hector partition applies only to systems where the total species pool has been sampled in monoculture, it is an example of a ‘population-level’ statistic (Figure 1a). In contrast, estimates derived from reduced subsets of species sampled from the total pool are known as ‘sample-level’ statistics (Figure 1b). In some cases, sample-level statistics can be used directly to approximate the population-level statistic. For example, because the sample-level mean approaches the population-level mean exactly as the sample size approaches that of the total population, we say that the sample-level mean is an ‘unbiased’ estimate of the population-level mean. However, not all statistics share this property. For example, because sample-level estimates of variance differ, on average, from population-level estimates by a factor of *N*/(*N* − 1), where *N* is the sample size, sample-level variance is usually multiplied by this factor before it is reported (i.e. uncorrected sample-level variance is a ‘biased’ estimate of population-level variance).

In this paper, we demonstrate how the classic population-level statistics for selection and complementarity effects can be estimated from an incomplete, random subsample of species. First, we show that uncorrected statistics calculated from sample-level observations are biased relative to the population-level values. Second, we derive a statistical correction that removes this bias. These estimates are prone to high error but can be made more precise by sampling a larger fraction of the community or a greater number of replicates. Finally, we apply our method using simulated data, and empirical observations from grassland communities in the Jena Experiment (Roscher et al., 2004; Weigelt et al., 2016; Weisser et al., 2017) and from nearby semi-natural grasslands (Buchmann et al., 2018).

## 2 Materials and Methods

### 2.1 Definition of classic complementarity and selection effects

Consider a community that contains a total of *Q* species, for which we wish to partition relationships between biomass measured in monoculture and in mixture. Following Loreau and Hector (2001), we define the ‘observed yield’ of species *i* in this mixture as *Y_i_*, and ‘expected yield’ as *M_i_*/*Q*, where *M_i_* is monoculture biomass. We then calculate ‘deviation in relative yield’ (i.e. difference between observed and expected relative yield) as

(1a) $$\Delta RY_{i}=\left(Y_{i}-\frac{M_{i}}{Q}\right)/M_{i}=\frac{Y_{i}}{M_{i}}-\frac{1}{Q}$$

Note that although we use *M_i_*/*Q* as the expectation for mixture yield, the derivations we describe below can be applied regardless of the null expectation chosen (although different null models may alter the estimated values of the statistics) (Loreau & Hector, in press). A primary contribution of Loreau and Hector (2001) was to show that the total deviation in yield (i.e. difference between observed and expected yield) calculated across all *Q* species can be expressed as follows:

(1b) $$\Delta Y=\sum_{i=1}^{N}\Delta RY_{i}M_{i}=N\bar{\Delta RY}\bar{M}+N\text{Cov}\;(\Delta RY,M),$$

where $\bar{X}=E[X]$ is the expected value of *X*. The terms on the far right-hand side of Equation 1b partition Δ*Y* into two components: a ‘complementarity effect’, *CE*, which is defined as

(1c) $$\text{CE}=N\bar{\Delta RY}\bar{M}$$

and a ‘selection effect’, *SE*, which is defined as

(1d) SE=NCov(ΔRY,M)

This derivation arises from the definition of covariance

(2) E(XZ)=XZ+Cov(X,Z)

In other words, the expected value of the product of any two random variables (regardless of their distribution, so long as they have means and variances) is equal to the product of their means, plus their covariance. Thus, Equation 1b is necessarily true for any sample of *Q* species.

### 2.2 Sample-level statistics

As defined in Equation 1c–d, *CE* and *SE* are population-level statistics. For clarity, we will refer to population-level statistics with superscript ‘*P*’ (e.g. *CE^P^* and *SE^P^*, calculated from a full sample of all *Q* species in the community), and sample-level statistics with superscript ‘*S*’ (e.g. *CE^S^* and *SE^S^*, calculated from a subset of *N* species drawn from a larger community of *Q* species).

Formal derivations of *CE^P^* and *SE^P^* as a function of *CE^S^* and *SE^S^* are available in Appendix A in the supplement. These yield the following approximations

(3a) $$\frac{1}{Q}{\text{SE}}^{P}≅\frac{1}{N}{\text{SE}}^{S}$$

(3b) $$\frac{1}{Q}{\text{CE}}^{P}≅\frac{1}{N}\left({\text{CE}}^{S}-\frac{1}{N}{\text{SE}}^{S}\right),$$

where the symbol ≅ indicates that these are unbiased approximations (i.e. they are distributed around the true values with some error). Rather than showing full derivations here, we instead include a simplified approach written in terms of ordinary least squares regression (OLS). Though less general than the technical derivation, this method may be more intuitive to many readers.

Imagine that we wish to characterize the relationship between two continuous variables *X* and *Z*. A simple way to do so is to assume that the two are related following a bivariate normal distribution with some covariance. Under these circumstances, if we observe a particular value of *X*, *X_i_*, we can write the expected value of *Z_i_* as

(4) $${\hat{Z}}_{i}|X_{i}=\bar{Z}+\frac{\text{Cov}\;(X,Z)}{\text{Var}\;(X)}(X_{i}-\bar{X}),$$

where $\bar{X}$ and $\bar{Z}$ are the mean values of *X* and *Z*, respectively. This parameterization is the ‘point-slope’ form of an OLS regression, with slope $\beta_{1}=\frac{\text{Cov}(X,Z)}{\text{Var}(X)}$ and intercept $\beta_{0}=\bar{Z}-\frac{\text{Cov}(X,Z)}{\text{Var}(X)}\bar{X}.$ Because Equation 4 is written purely in terms of means, variances and covariances, we can substitute in values for Δ*RY* and *M* from Equation 1c–d to characterize their relationship, yielding

(5a) $$\Delta \hat{R}Y_{i}|M_{i}=\bar{\Delta RY}+\frac{\text{Cov}\;(\Delta RY,M)}{\text{Var}\;(M)}(M_{i}-\bar{M})$$

By substituting in Equation 1c–d, we can rewrite Equation 4 in terms of *CE* and *SE* as

(5b) $$\Delta \hat{R}Y_{i}|M_{i}=\frac{\text{CE}}{N\bar{M}}+\frac{\text{SE}}{N\text{Var}\;(M)}\left(M_{i}-\bar{M}\right)$$

Thus, we can write *CE* and *SE* in terms of OLS regression parameters as

(5c) $$\text{CE}=N\bar{M}\left(\Delta \hat{R}Y_{i}|\bar{M}\right)=N\bar{M}\;(\beta_{0}+\beta_{1}\bar{M})$$

(5d) $$\text{SE}=N\beta_{1}\text{Var}\;(M)$$

In words, *CE* is just the number of species, times the mean monoculture yield, times the deviation in relative yield expected from the fitted regression at $M_{i}=\bar{M},$ while *SE* is the slope of the regression line, times the variance in monoculture yield, times the number of species.

**Figure 2:**
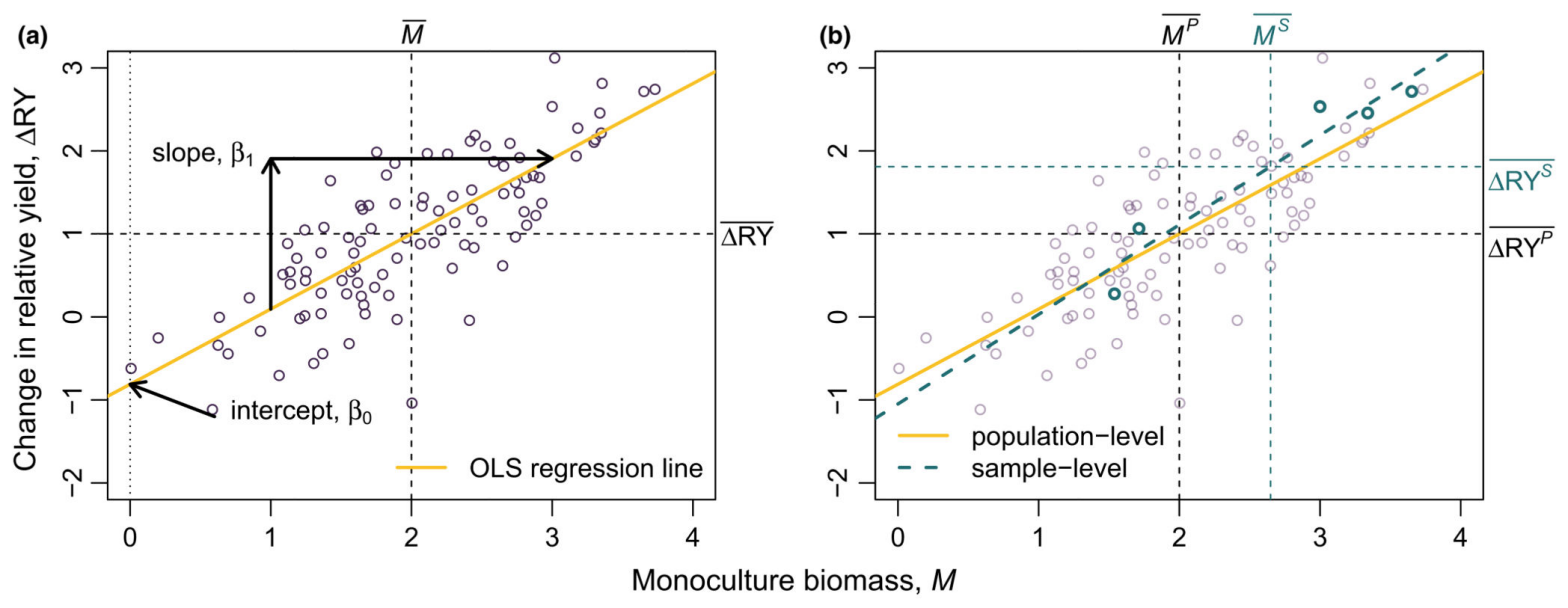
Visualizing the classic Loreau and Hector partition in terms of ordinary least squares (OLS) regression between monoculture biomass, *M*, and deviation in relative yield, Δ*RY*. Each point represents a single species. As shown in Equation 5a–d, the slope of the regression relates to selection effects, whereas complementarity effects correspond to the product of mean *M* and Δ*RY* observed across species (black dashed lines). If we compare the ‘population-level’ statistics estimated from fully sampling all 100 species in the community (a) versus the ‘sample-level’ statistic estimated from a random sample of five species drawn from the full community (points shown in green) (b), we find bias in the estimate of complementarity effects. This bias occurs because of covariance between *M* and Δ*RY*, which causes deviations between their sample-level and population-level means to be correlated

We can use this correspondence between OLS and the partition to visualize differences between sample-level and population-level statistics. Imagine a large population of *Q* species, for which *M_i_* and Δ*RY_i_* are known for all species (i.e. the monoculture biomass of each species, and the deviation in relative yield when grown in a mixture of all *Q* species). Plotting *M_i_* versus Δ*RY_i_* reveals a constrained relationship (Figure 2a), from which we can calculate *CE^P^* and *SE^P^* as a function of means and covariance, as shown in Equation 1a–d, or as a function of the OLS regression statistics, as show in Equation 5a–d.

Now, imagine that we were to sample only *N* species from the full population of *Q* species, and attempt to estimate *SE* and *CE* for the full community. By definition, if *SE* ≠ 0, we know that there is some covariance between *M_i_* and Δ*RY_i_*. For example, in Figure 2, we have *SE* > 0, and therefore positive covariance (n.b. *SE* < 0 would lead to similar results, but with a negative slope). Thus, we would also find positive covariance between sample-level estimates $\bar{M^{S}}$ and $\bar{\Delta RY^{S}}$ (Figure 2b).

**Figure 3:**
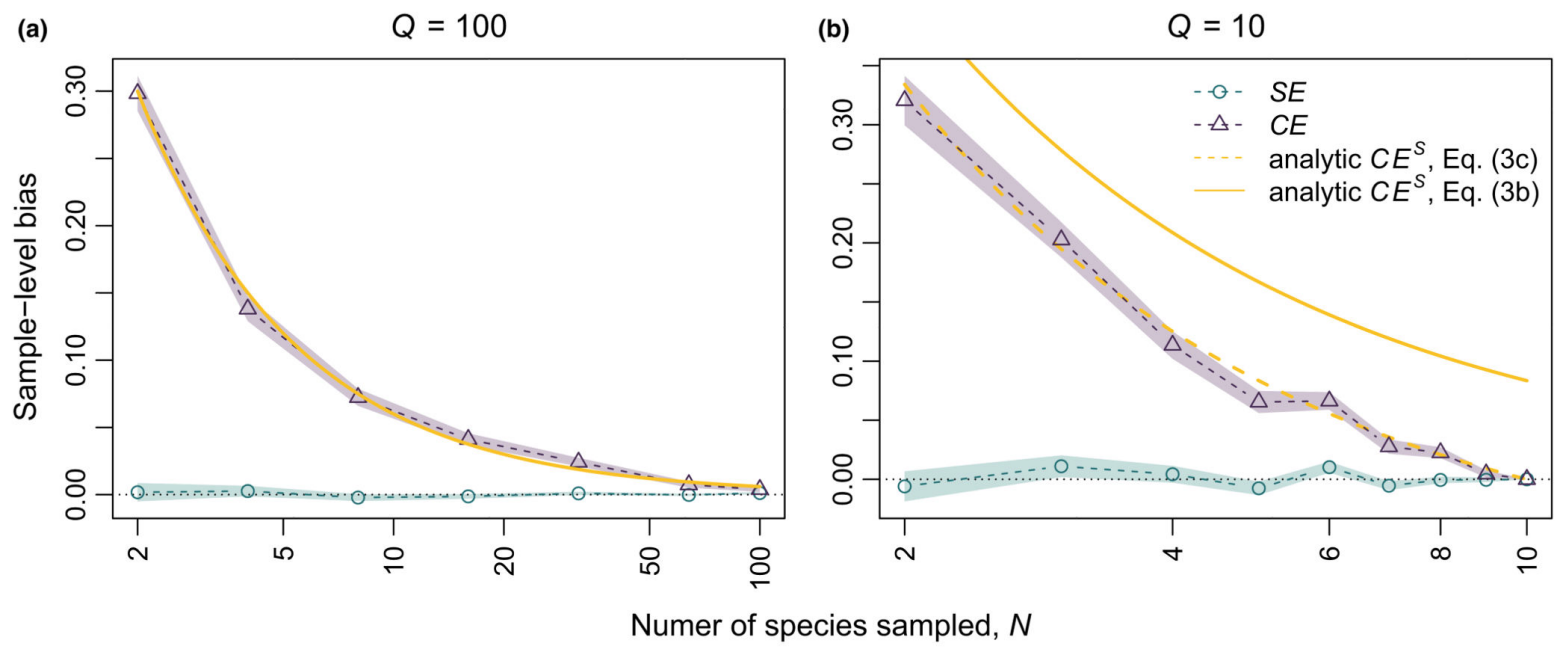
Deviation between the population-level and sample-level estimates of complementarity effects, *CE*, and selection effects, *SE* for two simulated communities. Points and shaded intervals show mean bias in the sample-level statistic, plus or minus one standard error of the mean observed across 20,000 random iterations. (a) For a large community (total species pool size *Q* = 100), bias as a function of the number of species sampled (*N*) can be predicted following Equation 3b. (b) For a smaller community (*Q* = 10), the bias is better estimated by Equation 3c, which accounts for the effects of finite *Q* (dashed yellow line), rather than Equation 3b (solid yellow line). For all comparisons, size of sample-level bias is shown as *SE^P^*/*Q* − *SE^S^*/*N*, or *CE^P^*/*Q* − *CE^S^*/*N*

Positive covariance between $\bar{M^{S}}$ and $\bar{\Delta RY^{S}}$ leads to an estimate of $E[\bar{M^{S}\Delta RY^{S}}],$ and thus of *CE^S^*, that is inflated relative to *CE^P^*, because $\bar{M^{S}}>\bar{M^{P}}$ implies $\bar{\Delta RY^{S}}>\bar{\Delta RY^{P}}.$ In contrast, the slope of the relationship between *M* and Δ*RY* is similar for both the population-level and the sample-level OLS, and *SE^S^* is thus equal to *SE^P^*. Following Equation 3b, we can use this estimate of *SE^P^* to correct our estimate of *CE^S^*, thus generating an unbiased estimate of *CE^P^* (Figure 3a). Strictly speaking, 1/*N SE^S^* ≅ 1/*Q SE^P^* only holds for sample-size corrected estimates of covariance, because like variance, sample-level covariance is biased relative to population-level covariance by a factor *N/*(*N* − 1). Because the sample-size corrected formula is the default method used by most software, understanding this distinction is probably not of great importance for most readers (see Appendix B for more details).

One note, regarding communities of small numbers of species (i.e. small *Q*). Under these circumstances, the analytical estimate of *CE^P^* in Equation 3b will be incorrect, because as *N* approaches *Q*, the difference between *CE^P^* and *CE^S^* must, by definition, approach zero (Figure 3b). The following correction can be applied to re-centre *CE^P^* around the true value

(3c) $$\frac{1}{Q}{\text{CE}}^{P}≅\frac{1}{N}\left({\text{CE}}^{S}-\frac{Q-N}{Q}\left(\frac{1}{N}{\text{SE}}^{S}\right)\right)$$

In general, we suggest Equation 3c over Equation 3b for all applications, as Equation 3c is more accurate for small *Q*, and differences between Equations 3b and 3c are negligible for large *Q*. Source code for calculating these population- and sample-level statistics are available in Appendix B in the supplement, and in the partitionBEFsp package in the R programming language (R Development Core Team, 2017).

### 2.3 Empirical examples

To test performance of our corrected statistics as a function of the number of sampled species (*N*), we use data collected from two systems: (a) experimentally assembled grassland plant monocultures and multi-species communities in the Jena Experiment (Roscher et al., 2004; Weisser et al., 2017); and (b) observational data from seminatural grassland communities located near the Jena Experiment (Buchmann et al., 2018). All analyses were conducted in R, version 3.5.2.

Plots in the Jena Experiment were established in 2002, and are weeded several times per year to maintain sown community composition. To facilitate analysis of the effects of observation error (see Appendix C), we use only data from May 2006, for which there was particularly high sampling intensity (four 20 × 50 cm^2^ samples in each multi-species mixture, and two 20 × 50 cm^2^ samples taken in each monoculture). Data are available in Weigelt et al. (2016). For the semi-natural grasslands, we consider two sites located about 0.1 km (site NwA) and 2.2 km (site GeA) from the Jena Experiment, respectively. The sites are mowed twice yearly in accordance with local management practices, but are otherwise unmanipulated. Species presence was estimated from six 80 × 80 cm^2^ quadrats in each field, and biomass was collected in May 2013 from 40 × 40 cm^2^ quadrants nested within the larger survey plots. Full results from surveys are reported in Buchmann et al. (2018), and data are available in Buchmann and Roscher (2019). For all sites, harvested biomass was sorted to species, and reported as dried mass in g/m^2^. See Appendix D for more details, and justification of the cross-year comparison.

We chose the two semi-natural grasslands because of their proximity to Jena, and because they span a broad range of species evenness (NwA is largely dominated by the grass species *Bromus erectus*, whereas GeA has high species evenness). Other studies have hypothesized that selection effects tend to be weakly or negatively correlated with evenness, whereas complementarity effects tend to be positively correlated with evenness (Hillebrand, Bennett, & Cadotte, 2008; Polley, Wilsey, & Derner, 2003). Thus, we expected to find weak selection and complementarity effects at NwA, and weak or negative selection effects and strong and positive complementarity effects at GeA.

We applied two empirical analyses. First, using data from the Jena Experiment, we calculated selection and complementarity effects for hypothetical subsamples of species drawn from the full community. We used data from all monocultures, but only two multi-species plots: one sown with eight species (i.e. *Q* = 8; plot B3A04), and one sown with 60 species (i.e. *Q* = 60, including 57 species with *M* > 0; plot B2A03). We chose these plots because they have particularly strong population-level selection effects relative to complementarity effects, which maximizes potential bias in *CE^S^* (i.e. they represent the ‘worst case scenario’ for our methods). For each multi-species plot, we sampled 20,000 random combinations of *N* species from the full community of *Q* species for all 2 ≤ *N* ≤ *Q*. For each random species combination, we calculated sample-level and expected population-level statistics for complementarity and selection effects using Equations 3a and 3c, and compared these to the expected values of the sample-level and population-level statistics calculated from the full pool of *Q* species. We then assessed the effects of sample size on uncertainty for *n* = 1, 10, or 30 ‘heterogeneous replicates’ (i.e. repeated draws of *N* randomly chosen species from the full pool of *Q* species – see Figure 1c,d).

Second, to demonstrate how our methods can be applied to naturally assembled systems, we calculated selection and complementarity effects for the two semi-natural grasslands, based on species-level biomasses in each site and monoculture data from the Jena Experiment. Field NwA included a total of 36 species, of which 26 were represented by monocultures (i.e. *Q* = 36, *N* = 26), whereas field GeA included 41 species, of which 33 were represented by monocultures (i.e. *Q* = 41, *N* = 33). Lastly, we tested how estimates varied as a function of species evenness, and compared these results to those from all four 60-species plots at Jena, which are the most similar to the semi-natural grasslands in terms of species richness (mean realized plot-level richness in the 60-species plots = 34.3 ± 2.2 *SD*; NwA = 24.8 ± 1.7 *SD*; GeA = 30.2 ± 4.1 *SD*; see Appendix E for more details).

## 3 Results

**Figure 4:**
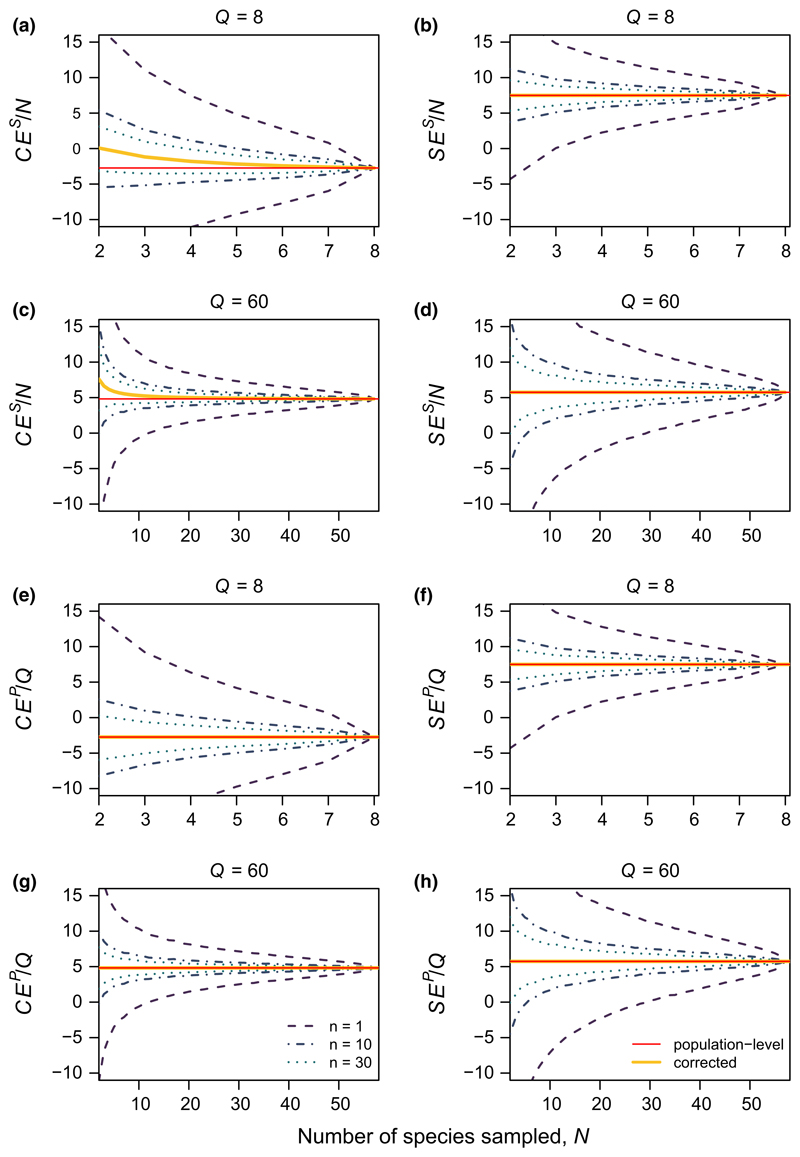
Sample-level versus population-level estimates of selection and complementarity effects from the Jena Experiment. For reference, red lines show the true population-level value. (a–d) Uncorrected sample-level estimates of complementarity effects (*CE^S^*) and selection effects (*SE^S^*) as a function of the number of sampled species (*N*) for grassland plant communities in the Jena Experiment. Panels (a–b) show results for a low-diversity community (*Q* = 8), and (c–d) for high-diversity (*Q* = 60). Solid yellow lines show the expected value of the sample-level statistic as a function of *N*, calculated from Equation 3a–c. Intervals show ±1 standard deviation of the mean calculated from 20,000 random draws of *N* species. Line styles show numbers of heterogeneous replicates (*n* = 1, 10, 30) (i.e. replicates of separate random draws of *N* species – see Figure 1b–c for example). (e–h) Corrected estimates of population-level complementarity effects (*CE^P^*) and selection effects (*SE^P^*) as a function of the number of sampled species (*N*) for grassland plant communities in the Jena Experiment, for the same low-diversity (e–f) and high-diversity communities (g–h). Intervals show variability in estimated population-level statistics as calculated from Equation 3a–c, and solid yellow lines show mean value calculated across simulations

For both the low and high diversity plots in the Jena Experiment, our results showed that incomplete samples of species provided unbiased, but noisy, estimates of sample-level and population-level complementarity effects and selection effects (Figure 4). As predicted in Equation 3a–c, uncorrected sample-level complementarity effects (*CE^S^*) differed from population-level complementarity effects (*CE^P^*) as an inverse function of *N* (Figure 4a,c). In contrast, *SE^S^* provided an unbiased estimate of *SE^P^* (n.b. Figure 4b vs. 4f, and 4d vs. 4h are identical). Though there was high variability across different combinations of species, variability dropped rapidly with *N*, especially for *N* > *Q*/2 or *N* > 10. Moreover, even for small *N*, moderate heterogeneous replication was sufficient to reduce uncertainty to manageable levels (Figure 1d).

**Figure 5:**
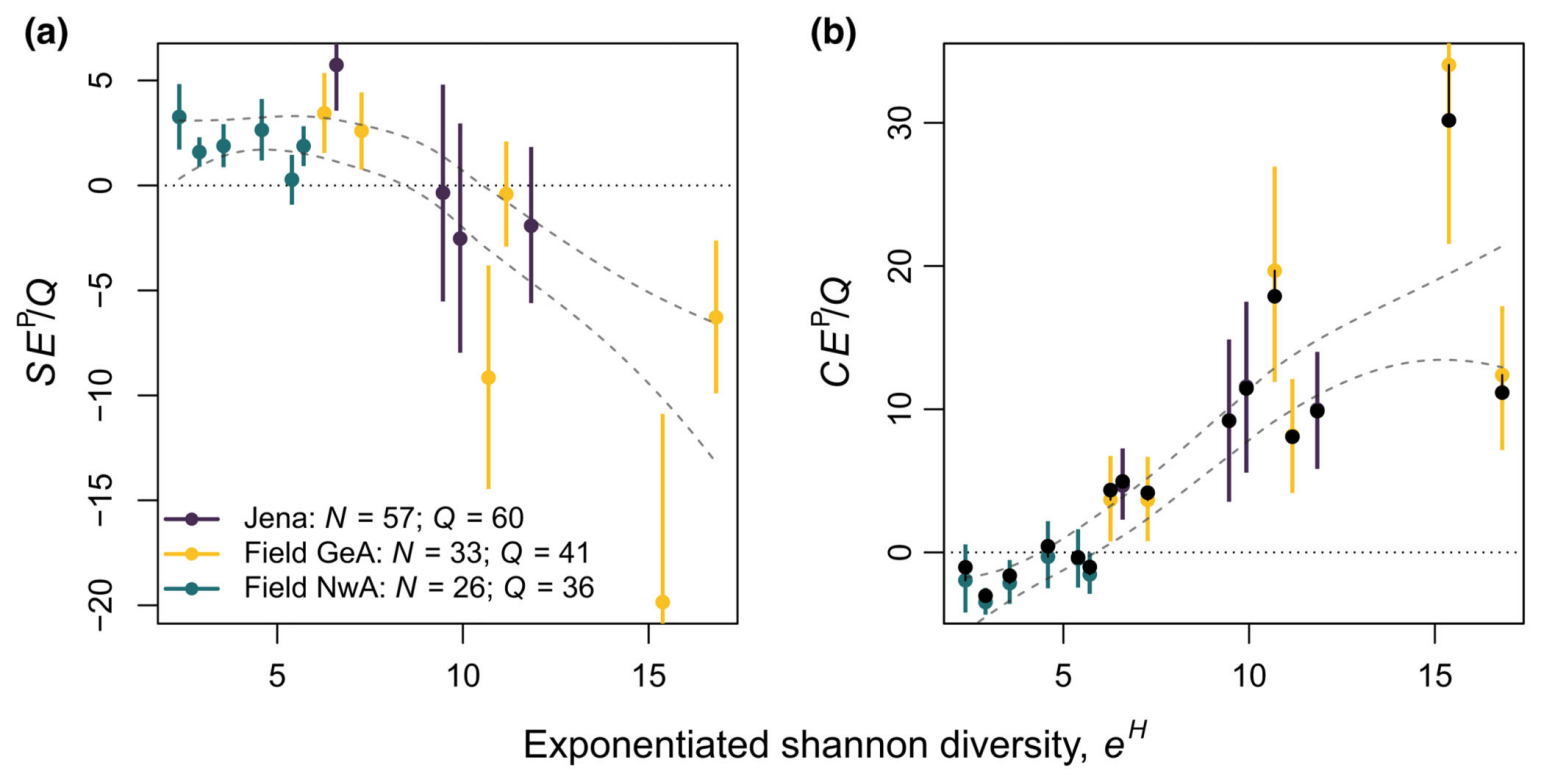
Estimated population-level (a) selection effects (*SE^P^*) and (b) complementarity effects (*CE^P^*) in the four 60-species plots in the Jena Experiment versus in six observational plots in each of two nearby semi-natural grasslands. Horizontal axis shows exponentiated Shannon diversity (*e^H^*). Coloured points and intervals show mean ± 1 standard deviation for each plot based on sampling error (i.e. error caused by incomplete sampling of the full community), following the methods described in Appendix E. Black points and lines in (b) show the difference between the uncorrected sample-level estimates of complementarity effects and the corrected population-level statistics, following Equation 3c. Dashed lines show mean ± 1 standard error of general trend across sites, based on a weighted loess regression

For the semi-natural grasslands, we found patterns that were largely consistent with expectations (Figure 5). For NwA, we found weakly positive selection effects and negative or zero complementarity effects. For GeA, selection effects decreased strongly with evenness, while complementarity effects increased strongly. Finally, for the 60 species plots at the Jena Experiment, results fell between those for NwA and GeA. Although sampling error was high, we still found significant differences in selection and complementarity effects among sites.

## 4 Discussion

Our results demonstrate that uncorrected sample-level selection and complementarity effects do not provide unbiased estimates of their classic population-level counterparts. More importantly, we identify statistical corrections that can be applied to remove this bias, thereby allowing estimation of selection and complementarity effects for communities even when measurements are only available for a random subset of species. The primary contributions of this paper are therefore the statistical estimates of *SE^P^* in Equation 3a, and *CE^P^* in Equation 3c.

### 4.1 Bias in sample-level estimates

Our findings suggest that uncorrected sample-level estimates will overestimate *CE^P^* given positive selection effects, and under-estimate *CE^P^* given negative selection effects. In contrast, estimates of *SE^P^* derived from incomplete samples are unbiased (again, given sample-size-corrected estimates of covariance – see Appendix B for details). Interestingly, most methods that extend the original Loreau and Hector partition have focused on selection effects (Fox, 2006; Isbell et al., 2018), suggesting that, at least from a statistical perspective, these finer partitions are also robust to bias due to incomplete sampling.

The magnitude of bias in uncorrected sample-level estimates of *CE^P^* is proportional to *SE^P^* (i.e. stronger selection effects lead to stronger bias) and is inversely proportional to *N* (i.e. the number of species sampled). Consequently, the bias declines rapidly with *N*. Similar reductions in bias also occur as *N* approaches *Q* (i.e. the total number of species in the community), because the sample-level statistic necessarily converges to the population-level statistic (Figure 4a,c). These relatively minor effects of bias suggest that our statistical correction is most important when *N* is small, especially for diverse communities (i.e. large *Q*). With stronger selection effects, however, larger *N* may be needed before uncorrected sample-level estimates of *CE^P^* approach the true value. As such, we suggest the use of the correction in Equation 3c even when *N* is large.

### 4.2 Sampling error and observation error

In contrast to bias, we find high sampling error in our estimates of *SE^P^* and *CE^P^* (i.e. although the estimates are centred around the true values, there is high uncertainty). We refer to this error as the ‘sampling error’, because it is a consequence of drawing incomplete samples of species from the full community (i.e. in contrast to ‘observation error’, discussed below). As with bias, sampling error declines strongly with *N* and *N*/*Q*. Additionally, however, sampling error can also be reduced through heterogeneous replication (i.e. increasing *n*; Figure 4). Note that heterogeneous replication is distinct from the type of replication that is typically carried out in experiments, in that it requires repeated draws of randomly chosen species (Figure 1b,c). For example, increasing heterogeneous replication might be accomplished by applying meta-analysis to compare multiple sites at which only partial monoculture data from distinct species pools are available (e.g. Hector et al., 1999), or by calculating average statistics across several different multi-species communities at a single site.

An important caveat for our results is that we assume that there is no observation error. Observation error refers to deviations between measured and true values – e.g. as might occur due to faulty equipment, incorrect species identifications, limited spatiotemporal scale of sampling, or effects of biological processes that are not accounted for in our methods (e.g. phenology). In other words, by ignoring observation error, we assume that biomass in monoculture (*M_i_*) and mixture (*Y_i_*) are known exactly for all *N* sampled species. In reality, however, observation error is often large in ecological studies (e.g. >20% of total observed variation). Because Δ*RY* is calculated as a ratio of mixture and monoculture biomasses, observation error in Δ*RY* is correlated with error in *M*, and the distribution of Δ*RY* is highly complex (Marsaglia, 2006) and difficult to assess analytically.

To partially address effects of observation error on our metrics, we include an additional simulation analysis of the Jena Experiment data in Appendix C in the supplement. In this analysis, we artificially add observation error to the data, based on variability observed among repeated samples that share the same species composition (e.g. replicate samples from within the same plot). Results again show that error is large, but can be controlled through moderate ‘homogeneous’ replication (i.e. repeated measurements of a single mixture; Figure 1d). Interestingly, for samples that include monocultures of a reasonably large proportion of the total community (e.g. *N* > *Q*/2), we find little difference in variability between estimates of *CE^P^* and *SE^P^* derived from our corrected sample-level observations, and those derived from observations of the full community (Figure S1 in the supplement). This result suggests that error due to incomplete sampling of the community (i.e. sampling error) may often be small relative to error due to imperfect observations (i.e. observation error).

### 4.3 Potential applications

To our knowledge, the statistics that we introduce are the first that allow unbiased estimation of selection and complementarity effects from incomplete samples of species. For example, in the analyses of the BIODEPTH experiment presented in the original Loreau and Hector (2001) paper, only about two-thirds of available multispecies mixture data were utilized, because monoculture data were lacking for some species (Hector et al., 1999). Our results suggest that for high diversity communities with monoculture data missing for only a few species (i.e. *N* ≈ *Q*), the effects of bias will be small, and uncorrected sample-level statistics will yield similar results to what would have been obtained by measuring the full population. For example, omitting a small number of species with zero biomass in monoculture, as is often done in analysis of biodiversity experiments (Marquard et al., 2009), will likely have minor impacts on the statistics.

As a general ‘rule of thumb’, our results suggest that reasonably stable estimates of *CE^P^* and *SE^P^* can be obtained given that at least half of the community has been sampled in monoculture (i.e. *N* > *Q*/2), even without replication (Figure 4, Figure S1). This finding has major implications for future biodiversity experiments, as it suggests that monocultures need not be maintained for all species, especially if total community size (i.e. *Q*) is large. Given the high maintenance cost of monocultures, planting a random subset of monocultures could free up resources for other experiments and tests (Loreau et al., 2001; Weisser et al., 2017). For example, maintaining multiple homogeneous replicates of a randomly chosen subset of monocultures and multi-species mixtures would help reduce uncertainty due to observation error (Cardinale et al., 2006; Schmid, Hector, Saha, & Loreau, 2008; Tilman et al., 1997). Additionally, for cross-site comparisons where experiments differ in their diversity treatments, the corrections suggested here may provide a way to ‘rarefy’ data from multispecies plots that differ in diversity (i.e. by estimating population-level statistics for a hypothetical community of shared size *Q*). While this approach would not control for biologically mediated changes in selection and complementarity effects, it would reduce differences that were due to statistical effects of community size.

The statistical corrections that we present here also facilitate calculation of complementarity and selection effects in naturally assembled systems. Such applications could be especially effective in leveraging information from existing biodiversity experiments to estimate complementarity and selection effects in nearby naturally assembled systems. For example, although monocultures in the Jena Experiment do not fully represent the species pool in the nearby sites that we test (Buchmann et al., 2018), it appears that they do contain a sufficiently large and random sample of the community to calculate stable estimates of selection and complementarity effects. Applications may be especially powerful in systems where experiments can be paired with nearby observational sites, such as the BEF China experiment (Bruelheide et al., 2011; Fichtner et al., 2017), or the ‘Big Biodiversity Experiment’ at Cedar Creek (Clark, Knops, & Tilman, 2019; Tilman et al., 1997). Similarly, however, these derivations may not be widely applicable in high diversity systems where monoculture data is not available for the large majority of species – e.g. in the neotropical forests near the Sardinilla biodiversity experiment, which contains monocultures of only six species (Potvin & Gotelli, 2008).

Applying our approach to natural systems may also be helpful in interpreting effects of non-random species loss on ecosystem functioning for the systems where it is applicable (Hooper et al., 2012; McGill, Dornelas, Gotelli, & Magurran, 2015; Pimm, Jones, & Diamond, 1988). If ecosystem functioning is largely driven by just a few highly influential species (i.e. strong selection effects, low complementarity effects), then the loss of locally rare species may have minor effects, whereas if ecosystem functioning is driven by synergistic effects of many different species (i.e. strong complementarity effects) then the non-random loss of rare species may have serious consequences for ecosystem functioning (Dee et al., 2019; Hillebrand et al., 2008; Polley et al., 2003). For example, in our analyses, selection and complementarity effects were small in natural systems that are dominated by a small number of species (field NwA), whereas in systems with high evenness (field GeA), both selection and complementarity effects were much larger and more variable than those observed in experimental data (Figure 5).

### 4.4 Caveats for subsequent applications

To help prevent misapplications of the methods that we introduce here, we offer a few words of caution. First, our bias correction formally applies to randomly chosen subsets of species. When subsamples are not random, estimates can be biased. In general, the size of this bias will be proportional to the degree of non-randomness in the subsample. If only a few species are missing, or if a large number of species are chosen from the community haphazardly, the bias should be small – but if monocultures only contain species that grow well under local conditions, or if large numbers of species fail to grown in monoculture, then the bias will be large. Our methods are therefore best suited to analyses of experimental data where random subsets of monocultures are chosen a priori, or in sites such as Jena where a large fraction of the natural community is represented in monocultures.

Second, when applying our method to natural communities, it is important to remember that the environmental conditions in the natural site may not be directly comparable to those in the experimental monocultures. If natural sites differ from the monocultures in systematic ways (e.g. higher soil fertility that leads to higher biomass among all species), then these differences could lead to misestimation of the complementarity effect. Conversely, if natural sites differ in ways that favour particular combinations of species, then these differences could influence estimates of selection effects. Results from our analyses must therefore either be interpreted under the assumption that initial conditions do not differ substantially between monoculture and mixture plots, or a more informative null model must be applied for calculating relative yield that takes these initial differences into account (e.g. by projecting expected monoculture yield under different conditions).

Finally, recall that the Loreau and Hector partition requires information about all species that make up the total species pool – including species that are unable to grow in multi-species mixtures. In our analyses, we attempt to address this problem by using regional surveys to determine the total species pool – thus, we included several species with zero observed biomass in mixture. However, this approximation is not entirely comparable to the conditions in experiments, where the full community that has been sown into a plot is known a priori. In general, care should be taken to include information from monocultures that represent the full suite of species that could potentially be present in a community. Failure to do so can lead to bias in both selection and complementarity effects.

### 4.5 Conclusions

Since its introduction in 2001, the Loreau and Hector partition has become one of the most influential and widely used statistics for studying relationships between biodiversity and ecosystem functioning. By extending this classic approach to cases where data are only available for a subset of the full community, we are optimistic that the corrected sample-level statistics that we present here will help facilitate broader and more correct application of these metrics. In particular, we hope that our methods will encourage more comprehensive use of existing experimental data, and estimation of selection and complementarity effects in natural systems.

## Supplementary Material

Supplementary Table 1

Supplementary Information 2

Supplementary Information

Supplementary Table 2
