# Supplementary material for: How to estimate complementarity and selection effects from an incomplete sample of species
Source: Methods Ecol Evol. Author manuscript; Available in PMC 2019 Dec 16. (PMC6914370; doi:10.1111/2041-210X.13285)
Supplement: Supplementary Information [file EMS85146-supplement-Supplementary_Information.pdf]

Supplement For:
How to Estimate Complementarity and Selection Effects from an Incomplete Sample of Species

Adam Thomas Clark*, *et al.*
**adam.tclark@gmail.com*

Contents:

Appendix A: Full derivation of population-level statistics

A.I: Selection effects

A.II: Complementarity effects

Appendix B: Source code for calculating population-level statistics

B.I: Description of files

B.II: Estimation of covariance

Appendix C: Effects of observation error

Appendix D: Study sites

D.I: Jena Experiment

D.II: Semi-natural grasslands

Appendix E: Example analysis of semi-natural grassland

E.I: Analytical methods

E.II: Confidence intervals

Appendix A: Full derivation of population-level statistics

A.I: Selection effects

From the definition of covariance, we know that the expected value of sample-level covariance is equal to the population-level covariance, such that $E[Cov(\Delta RY^S, M^S)] = Cov(\Delta RY^P, M^P)$. We can therefore express the relationship between sample-level and population-level selection effects as

$$SE^P = Q\ \mathrm{Cov}(\Delta \mathrm{RY}^P, M^P) = Q\ \mathrm{E}[\mathrm{Cov}(\Delta \mathrm{RY}^S, M^S)] = \frac{Q}{N}\mathrm{E}[SE^S] \cong \frac{Q}{N}SE^S \qquad \text{(S1a)}$$

and thus

$$\frac{1}{Q}SE^P \cong \frac{1}{N}SE^S \qquad \text{(S1b)}$$

where $X = E[X]$ is the expected value of random variable X, and the symbol ≅ indicates that $Q/N\ SE^S$ is an unbiased estimate of SE^P (i.e. the average value is distributed around the population-level value, with some error).

As discussed in the main text, note that sample-level covariance only provides an unbiased estimate of population-level covariance if it has been corrected for sample size (i.e. scaling by $N/(N\text{-}1)$). Because the sample-size corrected formula is used by default in most computerised methods, this difference is probably not a major concern for most users. Please see *Appendix B* for more details.

A.II: Complementarity effects

As noted in Eq. (2) in the main text, we know from the definition of covariances that the expected value of the product of two random variables is equal to the product of their expected values, plus their covariance. Thus, although $\overline{\Delta \mathrm{RY}^P}\ \overline{M^P} = \mathrm{E}[\overline{\Delta \mathrm{RY}^S}]\ \mathrm{E}[\overline{M^S}]$, we find

$$\mathrm{E}[\overline{\Delta \mathrm{RY}^S M^S}] = \mathrm{E}[\overline{\Delta \mathrm{RY}^S}]\ \mathrm{E}[\overline{M^S}] + \mathrm{E}[\mathrm{Cov}(\overline{\Delta \mathrm{RY}^S}, \overline{M^S})] \qquad \text{(S2a)}$$

and thus

$\overline{\Delta \text{RY}^P}\ \overline{M^P} = \text{E}[\overline{\Delta \text{RY}^S}\ \overline{M^S}] - \text{E}[\text{Cov}(\overline{\Delta \text{RY}^S}, \overline{M^S})]$ (S2b)

In other words, because the deviations between $\overline{M^S}$ and $\overline{\Delta \text{RY}^S}$ and their corresponding population-level means are correlated, these deviations leads to a systematic bias, proportional to their covariance.

To derive $\text{E}[\text{Cov}(\overline{\Delta \text{RY}^S}, \overline{M^S})]$, we follow a proof posted by *Stackexchange* user *Glen_b*, reproduced here for archival purposes (Glen_b, 2013). Recall that ΔRY^S and M^S are correlated only in a "pairwise" fashion – i.e. given two species *i* and *j*, ΔRY_i^S is correlated with M_i^S, and ΔRY_j^S is correlated with M_j^S, but ΔRY_i^S is independent of M_j^S, such that $Cov(\Delta RY_i^S, M_j^S) = 0$ if $i \neq j$. By definition, we can express this expected value as

$\text{E}\left[\text{Cov}\left(\overline{\Delta \text{RY}^S}, \overline{M^S}\right)\right] \cong \text{Cov}\left(\overline{\Delta \text{RY}^S}, \overline{M^S}\right)$ (S3a)

which we can expand as

$$= \text{Cov}\left(\frac{1}{N}\sum_i \Delta \text{RY}_i^S, \frac{1}{N}\sum_j \overline{M_j^S}\right) = \frac{1}{N^2}\sum_i \sum_j \text{Cov}\left(\Delta \text{RY}_i^S, M_j^S\right) \quad \text{(S3b)}$$

Lastly, since we know that $\text{Cov}\left(\Delta \text{RY}_i^S, M_j^S\right) = 0$ for all $i \neq j$, and that $\text{Cov}\left(\Delta \text{RY}_i^S, M_j^S\right) \cong \text{E}[\text{Cov}(\Delta \text{RY}^S, M^S)]$ for $i = j$, we can rewrite Eq. (S2b) as

$$= \frac{1}{N^2} N\text{Cov}(\Delta \text{RY}^S, M^S) = \frac{1}{N}\text{Cov}(\Delta \text{RY}^S, M^S) \quad \text{(S3c)}$$

Thus, by substituting Eq. (S3c) into (S2b), we can write the relationship between sample-level and population-level complementarity effects as

$$CE^P = Q\overline{\Delta \text{RY}^P}\ \overline{M^P} = Q\left(\text{E}\left[\overline{\Delta \text{RY}^S}\right]\text{E}\left[\overline{M^S}\right]\right) \quad \text{(S4a)}$$

$$\cong Q\left(\overline{\Delta \text{RY}^S}\ \overline{M^S} - \frac{1}{N}\text{Cov}(\Delta \text{RY}^S, M^S)\right) \cong \frac{Q}{N}\left(CE^S - \frac{1}{N}SE^S\right)$$

and thus

$$\frac{1}{Q}CE^P \cong \frac{1}{N}\left(CE^S - \frac{1}{N}SE^S\right) \quad \text{(S4b)}$$

Note that Eqs. (S4a-b) imply that, although an unbiased estimate of CE^P can be obtained from the sample-level complementarity effect, CE^S must first be corrected by a factor of $1/N\ SE^S$. In other words, unlike selection effects, the raw sample-level metric for the complementarity effect is not, on its own, an unbiased estimate of the population-level complementarity effect.

Appendix B: Source code for calculating population-level statistics

B.I: Description of files

All functions needed to apply the analyses in the main text are available in the `partitionBEFsp` package, written for the `R` programming language (R. Development Core Team, 2017). The file `partitioning_functions.R` includes a copy of these functions. These can be used for estimating: (1) the classic selection and complementarity effects, *sensu* Loreau and Hector (2001); (2) population-level statistics based on an incomplete sample of *N* species from a larger community of *Q* species; and (3) the expected value of the sample-level statistics for a sample of *N* species drawn randomly from a community of *Q* species. The package and script file also includes an example script showing how to apply these functions.

B.II: Estimation of covariance

One important note about the functions we provide here is that they include three potential methods for calculating covariance. Recall from the introduction that we discussed that the sample-level variance is a biased estimate of the population-level variance, which is why sample variance is typically calculated as $\mathrm{var}(X) = \sum((X-\bar{X})^2)/(N-1)$. A similar bias occurs for sample-based estimates of covariance, such that $\mathrm{cov}(X,Y) = \sum\big((X-\bar{X})(Y-\bar{Y})\big)/(N-1)$ for sample-level estimates.

Given the default argument `uncorrected_cov = FALSE`, we use the standard "`cov`" function from R, which applies the sample-size correction and assures that $SE^P \cong SE^S$. This approach is probably the correct one to use for most applications, and is the approach that we apply in all analyses presented in this manuscript. However, one drawback to this method is that $CE + SE$ is no longer guaranteed to equal the true deviation in yield ΔY. In

general, this difference is probably of minor importance, since estimates if *SE* are typically used to determine the direction of the association between monoculture and mixture yields, rather than to precisely calculate the change in yield itself (n.b. this difference is also the reason why CE^S can be a biased estimate of the population-level statistic, while SE^S is not, even though their sum is theoretically equal to ΔY).

Nevertheless, we also include the option `uncorrected_cov = TRUE`, which applies the non-sample-size-corrected formula $\mathrm{cov}(X, Y) = \sum\left((X - \bar{X})(Y - \bar{Y})\right)/N$. This implementation guarantees that $CE + SE = \Delta Y$ for any mixture of species and is potentially useful for testing that the functions have been written correctly. However, it should probably not be applied in most analyses, as it no longer guarantees that $SE^P \cong SE^S$ (especially for small *N*).

Finally, we include a compromise function, `uncorrected_cov = "COMP"`, which applies an augmented correction similar to that in in Eq. (3c) in the main text: $\sum\left((X - \bar{X})(Y - \bar{Y})\right)/(N - \frac{Q-N}{Q})$. This formula ensures that as *N* approaches *Q*, SE^S approaches the "true" SE^P that would have been realised had the sample-size correction not been applied, such that $SE^{S=P} + CE^{S=P} = \Delta Y$. However, the correction also ensures that $SE^P \cong SE^S$ for small *N*. In general, if *Q* is large, this formula should yield similar results to the default `uncorrected_cov = FALSE`. Again, we do not recommend applying this procedure except in cases where it is vital that the sum of the selection effects and complementarity effects equals the change in relative yield.

Appendix C: Effects of observation error

Our primary analyses in the main text assume that we perfectly know the true monoculture and mixture biomass of species (i.e. that we can estimate M_i and Y_i without error). In reality, however, observation error is typically high in ecological studies. To demonstrate the effects of observation error, we repeated our analysis of the high diversity mixtures from the Jena Experiment, but with the addition of simulated observation error.

For each of 20,000 iterations, we took the mean values for M_i and Y_i for each species and added random noise (drawn from a log-normal distribution), based on between-sample variability observed for each species in the Jena Experiment. For simplicity, we included both variability among plots and among replicate samples within plots, meaning that our "observation error" estimates contained aspects of both measurement error and of spatial heterogeneity (n.b. this interpretation of observation error is typical of most grassland biodiversity experiment designs, as the location from which samples are taken moves from year to year to prevent long-term effects of vegetation clipping from influencing results). For species where there was insufficient data to calculate variability, we used the mean variability observed across all other species.

For each of these 20,000 simulated iterations of noisy observations of M and Y, we calculated two types of estimates of CE^P and SE^P. First, in order to quantify the effect of observation error, we used the full pool of Q species to calculate the classical complementarity and selection effect metrics (yellow lines in Fig. S1). Second, in order to show how our sample-level approximations of the population-level statistics were influenced by observation error, we estimated CE^P and SE^P based on incomplete samples of N species drawn from the full pool of Q species (dark blue lines in Fig. S1). Thus, these sample-level estimates were influenced by both observation error and sampling error (i.e. inaccuracies due to only partially sampling the full community of species). Lastly, for both types of metrics,

we calculated variability across iterations as a function of the number of homogeneous replicates (m = 1, 10, 30). Unlike "heterogeneous replicate" number, which we call n in the main text, "homogeneous replicate" number m represents replication of identical mixtures of species (e.g. m replicated observations of a fixed set of N species). See Fig. 1b-c in the main text for example.

In general, we found that observation error led to high uncertainty, but that this uncertainty could be effectively controlled with realistic numbers of homogeneous replicates (Fig. S1). Though error in the sample-level approximations of CE^P and SE^P were (by necessity) always higher than those estimated from the full pool of Q species, the difference declined rapidly with N. Thus, for $N > Q/2$ (i.e. $N \approx 30$), variability in the sample-level approximations was roughly equal to that from the estimates derived from the full pool of Q species. This result suggests that for reasonable levels of observation error and sample sizes of at least $N = Q/2$, uncertainty in the sample-level estimates is small relative to that caused by observation error.

Appendix D: Study sites

D.I: Jena Experiment

The Jena Experiment is a large grassland biodiversity experiment established on a formerly highly fertilized agricultural field in 2002 (Roscher et al., 2004). The experimental site is located on the floodplain of the river Saale near to the city of Jena (Thuringia, Germany; 50°55' N, 11°35' E, 130 m a.s.l.). The soil is a Eutric Fluvisol and soil texture changes from sandy loam to silty clay with increasing distance to the river. The experimental species pool consists of 60 species common to Central European grasslands, which were classified into four functional groups (grasses = 16 species, legumes = 12 species, small herbs = 12 species, tall herbs = 20 species). Plant communities were designed by realizing all possible combinations of a gradient in species richness (1, 2, 4, 8, 16 and 60 species) crossed with a gradient in functional group number (1, 2, 3, and 4 functional groups).

All species-richness levels were established with 16 “heterogeneous” replicates (i.e. replicates could differ in composition), except for the 16-species mixtures which included 14 heterogeneous replicates (because pure legume and small herb mixtures with 16 species were not possible), and for the 60-species mixture which included four “homogeneous” replicates (i.e. all plots shared the same species composition), resulting in a total of 82 plots of 20 × 20 m size. Mixture compositions were randomly created from the respective functional groups. In addition, all 60 species were established in replicated monocultures of 3.5 × 3.5 m size. Plant communities were established by sowing with a total density of 1000 viable seeds per m^2 with equal proportions of each species in the mixtures. To control for variation in soil properties as the distance to the Saale river increases, the experiment was established in four blocks parallel to the river.

All plant communities are weeded two or three times per year to maintain the sown species combinations. Plots are mowed two times per year (early June, early September) and

mown plant material is removed as usual for extensively managed hay meadows in the region. Plots do not receive any fertilizer. Aboveground biomass is harvested twice per year shortly before mowing in late May and late August. For our case study, we used biomass data from the first harvest, which usually represents peak biomass (sampled in 2006). Biomass was harvested in rectangles of 50 × 20 cm size by cutting plant material 3 cm above soil surface. Four and two randomly distributed samples were taken in each large and small plot, respectively. Biomass samples were sorted to sown species, weeds and detached dead material, dried at 70°C for at least 48 h and weighed.

D.II: Semi-natural grasslands

The studied semi-natural grasslands were old permanent grasslands, which are managed by mowing two times per year without fertilization, as in the Jena Experiment plots. One study site (NwA) is situated directly beside the Jena Experiment area (0.1 km distance), while the other site (GeA) is located in 2.2 km distance (Buchmann et al. 2018). On each site, a plot of 7.5 × 7.5 m was established, consisting of a grid of 36 subplots of 0.8 × 0.8 m size separated by paths of 0.5 m width. According to the results of species inventories in August 2012, six subplots representing the lowest, intermediate, and highest species richness on each site were chosen. In the centre of these subplots, aboveground biomass was harvested in a quadrat of 40 × 40 cm at estimated peak biomass in late May 2013. Plant material was cut at 3 cm above soil surface, sorted to species and detached dead material and weighed after drying at 70°C for 48 h. Species inventories were repeated in August 2013 on the subplot area of 80 × 80 cm size and summarized with species occurrences in the biomass samples to estimate species richness for each subplot.

Note that for our comparisons of Jena vs. these semi-natural grasslands, we use two different sets of sample years. We justify this inter-year comparison in three ways. First,

inter-year comparisons will likely be necessary for most studies that seek to apply our methods, as long-term annual data on monocultures is rarely available (e.g. more commonly, historical monoculture data might be available from a site, and compared to multi-species mixture yields measured in other studies carried out in subsequent years). Thus, we aimed to demonstrate that stable estimates of selection and complementarity effects are still possible, even when comparing data from across years. Similarly, the 2006 data from the Jena Experiment are probably more representative of the kinds of monoculture information that are most likely to exist for other sites and systems than are data from later years (i.e. although longer-term data do exist from the Jena Experiment, multi-decade experimental studies are very rare). Finally, although weather differed somewhat between 2006 vs. 2013 (e.g. over the early growing season March-May, total precipitation at the weather station of the Jena Experiment site in the two years was 148.8 mm vs. 223.4 mm, and average temperature was 8.0°C vs. 6.8°C, respectively), we still found a gradient of selection and complementarity effects across sites, with plots from the Jena Experiment falling roughly in the middle of the gradient. This gradient suggests that our results were not dominated by year-to-year changes in mean biomass, which would have resulted in complementarity effects that were similar between the two semi-natural sites, but that differed greatly from the Jena Experiment (i.e. because of coordinated changes in ΔRY between the two semi-natural sites). Note that although there was major flooding around Jena in 2013, most of the precipitation related to this event fell after the biomass harvest in May.

Appendix E: Example analysis of semi-natural grassland

E.I: Analytical methods

To calculate population-level selection and complementarity effects (SE^P and CE^P, respectively) for the two semi-natural grasslands near Jena, we used similar methods to those applied in the Jena Experiment. First, based on presence-absence data from the larger 80 x 80 cm plots in each site, we determined the "total species pool" for each location. Because the total species pool is meant to represent total potential membership in the community (rather than just recording presence of species with nonzero biomass in mixture), we pooled all six plots in each site to estimate this species pool, yielding $Q = 41$ for site GeA and $Q = 36$ for site NwA. We then matched this list of species to the full list of monoculture plots in the Jena Experiment, which were used to estimate monoculture biomass for both sites. Of the full community at GeA, monocultures for $N = 33$ species were available; for NwA, monocultures for $N = 26$ were available.

Species abundance in mixture was calculated for each of the six plots at each site, based on sorted species-level biomass measured in the 40 x 40 cm plots nested within the larger 80 x 80 cm plots, and reported in dried gm^{-2}. In addition to using these values as indicators of mixture biomass for our calculations of selection and complementarity effects, we also used these species-level abundances to calculate exponentiated Shannon Diversity in each plot, as $e^H = \exp(-\Sigma\, p_i \log(p^i))$, where p_i is the relative abundance of species i.

E.II: Confidence intervals

To estimate the confidence intervals for SE^P and CE^P shown in Fig. 5 in the main text, we proceeded in two steps. First, for each plot in each site, we parameterised a bivariate normal distribution based on the relative yield difference (ΔRY_i) and the mean monoculture

abundance (M_i) observed across all species, the variance of ΔRY_i and of M_i observed across all species, and the covariance of ΔRY_i vs. M_i. We then re-sampled N species from this distribution 20,000 times for each plot, and estimated SE^P and CE^P for each iteration following Eqs. (3a) and (3c) in the main text. Finally, we calculated the mean and standard deviation of these statistics across all 20,000 iterations. Note that this procedure guaranteed that the means value of these distributions perfectly matched the values generated by calculating SE^P and CE^P directly from observed ΔRY_i and M_i.

References

Buchmann, T., Schumacher, J., Ebeling, A., Eisenhauer, N., Fischer, M., Gleixner, G., … Roscher, C. (2018). Connecting experimental biodiversity research to real-world grasslands. *Perspectives in Plant Ecology, Evolution and Systematics*, *33*, 78–88. doi:10.1016/j.ppees.2018.06.002

Buchmann, T., & Roscher, C. (2019). Data from: How to estimate complementarity and selection effects from an incomplete sample of species. *Dryad Digital Repository*, https://doi.org/10.5061/dryad.26k70d6

Glen_b. (2013). Estimating the covariance of the means from two samples. Retrieved 16 December 2018, from https://stats.stackexchange.com/questions/59546/estimating-the-covariance-of-the-means-from-two-samples

Loreau, M., & Hector, A. (2001). Partitioning selection and complementarity in biodiversity experiments. *Nature*, *412*, 72–76.

R. Development Core Team,. (2017). R: a language and environment for statistical computing (Version 3.4.2). R Foundation for Statistical Computing.

Roscher, C., Schumacher, J., Baade, J., Wilcke, W., Gleixner, G., Weisser, W. W., … Schulze, E.-D. (2004). The role of biodiversity for element cycling and trophic interactions: an experimental approach in a grassland community. *Basic and Applied Ecology*, *5*(2), 107–121. doi:10.1078/1439-1791-00216

Weigelt, A., de Luca, E., Roscher, C., Temperton, V., Buchmann, N., Fischer, M., … Meyer, S. T. (2016). *Collection of aboveground community and species-specific plant biomass from the Jena Experiment (time series since 2002).* PANGAEA. doi:10.1594/PANGAEA.866358

Supplementary Tables:

Table S1: Presence/absence data for the semi-natural grasslands, from the large 0.8 x 0.8 m plots used to determine community composition. Data are from Buchmann et al. (2018). Species abbreviations match those used in the Jena Experiment by Weigelt et al. (2016). Data can be downloaded from Dryad, at DOI 0.5061/dryad.26k70d6 (Buchmann & Roscher 2019).

Table S2: Aboveground biomass data for the semi-natural grasslands, from the smaller 0.4 x 0.4 m plots used to determine species abundance in mixture. Reported in gm^{-2} dried mass. Data are from Buchmann et al. (2018). Species abbreviations match those used in the Jena Experiment by Weigelt et al. (2016). Data can be downloaded from Dryad, at DOI 0.5061/dryad.26k70d6 (Buchmann & Roscher 2019).

Supplementary Figures:

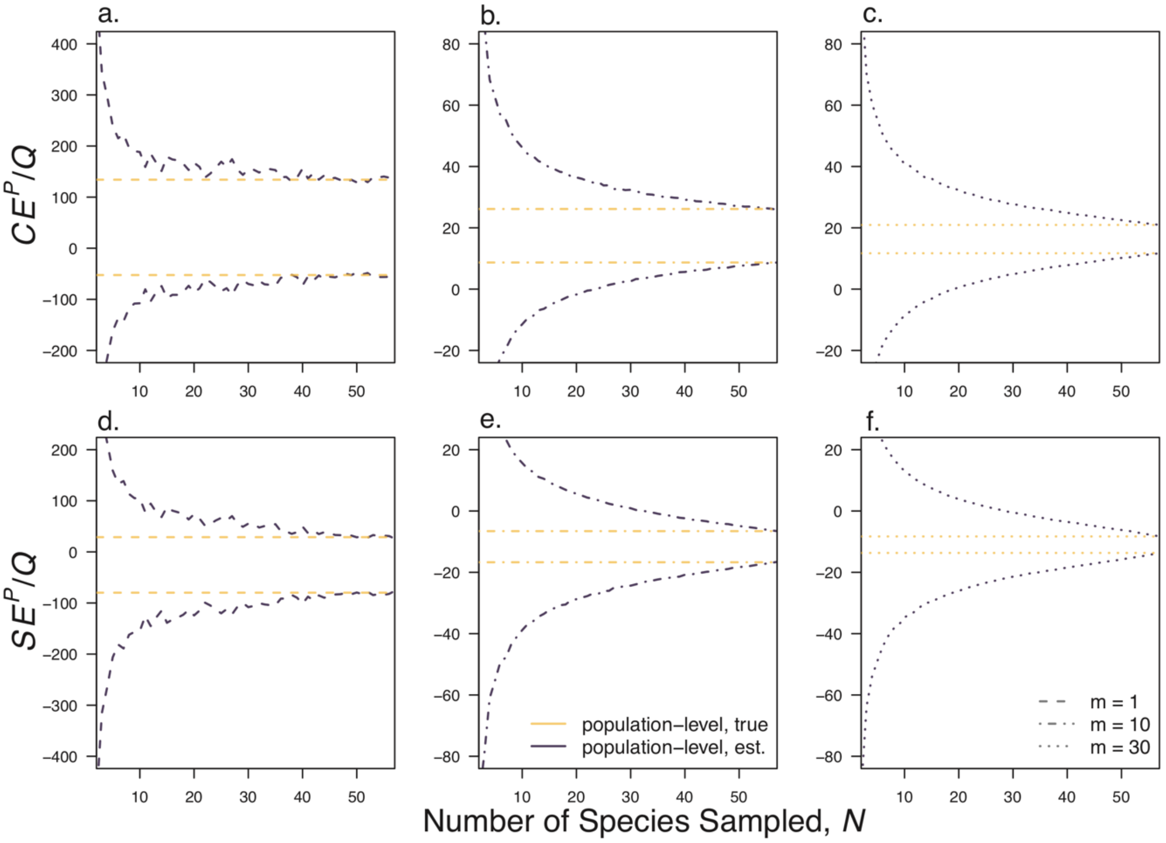

Figure S1: Effects of observation error on estimates of population-level statistics for complementarity effects **(a-c)** and selection effects **(d-f)**, based on the Jena grassland plant community shown in Figs. 4c-d and 4g-h in the main text (i.e. $Q = 57$). Columns differ in number of "homogeneous" replicates, denoted by line styles ($m = 1, 10, 30$) (see Fig. 1c-d in the main text for example of replication methods). Intervals show ± one standard error of the mean from 20,000 random draws of species. Yellow intervals show variability in the population-level statistics calculated from the full pool of Q species – i.e. variability is purely a result of observation error. Dark blue intervals show estimates of population-level effects based on finite samples of N species following Eqs. (3a-c) in the main text – i.e. variability is a result both of uncertainty in the true composition of species in the full community, as well as observation error. Panels **(a,d)** are shown on an extended y-axis for clearer visualization.
